# 26.3 SALIENCE SIGNALING AND THE EMERGENCE OF PSYCHOPATHOLOGY IN YOUTH AT CLINICAL HIGH RISK FOR PSYCHOTIC ILLNESS

**DOI:** 10.1093/schbul/sby014.108

**Published:** 2018-04-01

**Authors:** James Waltz, Caroline Demro, Zachary Millman, Gloria Reeves, Jonathan Roiser, James Gold, Jason Schiffman

**Affiliations:** 1 Maryland Psychiatric Research Center; 2 University of Maryland, Baltimore County; 3 University of Maryland School of Medicine; 4 UCL Institute of Cognitive Neuroscience

## Abstract

**Background:**

The early identification of people who appear to be at high risk for conversion to psychosis has become a central thrust of mental health research, with the hope that early intervention may alter the course of psychotic illness. Importantly, both positive and negative symptom dimensions have been found relate to risk for conversion in clinical high risk (CHR) populations. Neuroimaging work points to a role for dopamine pathway activity in both the positive and negative symptoms of psychotic illness. A role for dopamine pathways in signaling various kinds of salience is well-established, and several authors have proposed that excessive dopamine transmission in the striatum might contribute to psychotic symptoms by bringing about erratic, or “aberrant”, salience signaling. By contrast, a reduced ability to identify salient events as such, and signal salience “adaptively”, could result in impairments in learning and motivation. I will describe results from a study in which we examined the impact of salient events on learning and behavior in adolescents and young adults, a subset of whom were identified as being at CHR for developing psychotic illness.

**Methods:**

Participants were 98 adolescents and young adults (mean age = 16.1 ± 3.3 years), assessed clinically using the Structured Interview for Psychosis-Risk Syndromes (SIPS). Eighty-nine participants were receiving mental health services, with 30 of the 89 identified as CHR and 8 identified as already having a psychotic illness. We used two experimental paradigms to investigate the impact of salient events on learning and behavior: the probabilistic stimulus selection task (PSST; Frank et al., 2004) and the Salience Attribution Task (SAT; Roiser et al., 2009). Both adaptive and aberrant salience signals were operationalized in the context of each task. Successful performance of the PSST depends on the adaptive signaling of mismatches between expected and obtained outcomes, called reward prediction errors, which are one form of salient event. The SAT requires participants to respond as quickly as possible to a response prompt, which is preceded by conditioned stimuli that potentially predict reward availability for a fast response. The comparison of reaction time (RT) between responses following the frequently vs. infrequently rewarded conditioned stimuli offers a measure of adaptive salience coding with the expectation of faster RT for reward predicting stimuli. The comparison of RT between responses to the two levels of the irrelevant dimension offers a measure of aberrant salience coding with the expectation of equal RT for stimuli equally-predictive of reward. We assessed whether experimental measures of both adaptive and aberrant salience showed correspondences with SIPS ratings for symptoms along both the positive and negative dimensions.

**Results:**

We observed significant correlations between multiple performance measures from the PSST and measures of both positive and negative symptoms. We found that positive symptom severity, in help-seeking youth, correlated positively with an implicit measure of aberrant salience from the SAT, and negatively with an explicit measure of adaptive salience.

**Discussion:**

These results, consistent with our previous findings in both first-episode psychosis patients and patients with chronic schizophrenia, suggest that experimental measures of salience signaling may provide a psychosis risk signal in treatment-seeking youth. Further research is necessary to understand the potential predictive role of these measures for conversion to psychosis.

